# Osteogenesis of Iron Oxide Nanoparticles-Labeled Human Precartilaginous Stem Cells in Interpenetrating Network Printable Hydrogel

**DOI:** 10.3389/fbioe.2022.872149

**Published:** 2022-04-29

**Authors:** Wei Liao, Jingwei Lu, Qianjin Wang, Sen Yan, Yan Li, Yibo Zhang, Peng Wang, Qing Jiang, Ning Gu

**Affiliations:** ^1^ Children’s Hospital of Nanjing Medical University, Nanjing, China; ^2^ School of Biomedical Engineering and Informatics, Nanjing Medical University, Nanjing, China; ^3^ Department of Orthopedics, Jinling School of Clinical Medicine, Nanjing Medical University, Jinling Hospital, Nanjing, China; ^4^ State Key Laboratory of Pharmaceutical Biotechnology, Department of Sports Medicine and Adult Reconstructive Surgery, Nanjing Drum Tower Hospital, The Affiliated Hospital of Nanjing University Medical School, Nanjing, China; ^5^ State Key Laboratory of Bioelectronics, Jiangsu Key Laboratory for Biomaterials and Devices, School of Biological Sciences and Medical Engineering, Southeast University, Nanjing, China; ^6^ Nanjing Drum Tower Hospital Clinical College of Nanjing Medical University, Nanjing, China

**Keywords:** iron oxide nanoparticles, human precartilaginous stem cells, osteogenic activity, interpenetrating network hydrogel, bone regeneration

## Abstract

Smart biomaterials combined with stem cell-based therapeutic strategies have brought innovation in the field of bone tissue regeneration. However, little is known about precartilaginous stem cells (PCSCs), which can be used as seed cells and incorporated with bioactive scaffolds for reconstructive tissue therapy of bone defects. Herein, iron oxide nanoparticles (IONPs) were employed to modulate the fate of PCSCs, resulting in the enhanced osteogenic differentiation potential both *in vitro* and *in vivo*. PCSCs were isolated from the ring of La-Croix extracted from polydactylism patient and identified through immunohistochemically staining using anti-FGFR-3 antibodies. Potential toxicity of IONPs toward PCSCs was assessed through cell viability, proliferation, and attachment assay, and the results demonstrated that IONPs exhibited excellent biocompatibility. After that, the effects of IONPs on osteogenic differentiation of PCSCs were evaluated and enhanced ALP activity, formation of mineralized nodule, and osteogenic-related genes expressions could be observed upon IONPs treatment. Moreover, *in vivo* bone regeneration assessment was performed using rabbit femur defects as a model. A novel methacrylated alginate and 4-arm poly (ethylene glycol)-acrylate (4A-PEGAcr)-based interpenetrating polymeric printable network (IPN) hydrogel was prepared for incorporation of IONPs-labeled PCSCs, where 4A-PEGAcr was the common component for three-dimensional (3D) printing. The implantation of IONPs-labeled PCSCs significantly accelerated the bone formation process, indicating that IONPs-labeled PCSCs could endow current scaffolds with excellent osteogenic ability. Together with the fact that the IONPs-labeled PCSCs-incorporated IPN hydrogel (PCSCs-hydrogels) was biosafety and printable, we believed that PCSCs-hydrogels with enhanced osteogenic bioactivity could enrich the stem cell-based therapeutic strategies for bone tissue regeneration.

## 1 Introduction

Repair of large bone defects caused by trauma, disease, or tumor resection has become a fundamental challenge in the field of orthopedics (Stahl and Yang, 2021). Currently, autologous or allogeneic bone grafts are considered as the most appropriate materials for the treatment of large bone defects but come with some limitations such as infection, possible fracture, and limited bone availability (Brink, 2021). To overcome these shortcomings, functional tissue engineering bone grafts, containing osteoblast or stem cells, growth factor, and bioactive materials, are considered as promising alternatives that have attracted a great deal of interest from researchers and focuses on regenerative strategies for large bone defects (Battafarano et al., 2021; Chansaenroj et al., 2021). Recently, stem cell-based therapeutic strategies have attracted considerable attention in the field of bone tissue regeneration due to their excellent osteogenic potential, superior biocompatibility, low immunogenicity, and ease of accessibility (Shang et al., 2021). In addition to their differentiation potential, stem cells also have ability to regulate other cells’ function and systemic inflammatory condition through cell–cell interaction to enhance their therapeutic efficacy (Sui et al., 2019). This was an advantage for bone repair using stem cell-based therapeutic strategies.

There are several kinds of stem cells, including embryonic stem cells (ESCs), bone marrow mesenchyml stem cells (BMSCs), and adipose-derived mesenchymal stem cells (ADSCs), which have been used as seed cells of tissue engineering for substitute therapies of bone defects (Kim et al., 2011; Pan et al., 2016; Du et al., 2021; Mende et al., 2021). Although the current stem cell-based therapeutic strategies exhibited potential capacity for bone tissue regeneration they still have some limitations in terms of difficulty in isolation, purification, and manipulation of stem cell fate. Apart from these stem cells, precartilaginous stem cells (PCSCs), a kind of adult stem cells that can be isolated from the peripheral layer of the epiphyseal organ with a perichondrial mesenchyme in embryo limbs (the ring of La-Croix), have strong proliferation ability and differentiation potential (You et al., 2011). There are accumulating evidence that PCSCs exhibit excellent chondrogenic activity and have been extensively utilized as seed cells for reconstructive tissue therapy of cartilage defects due to their excellent chondrogenic ability (Guo et al., 2013; Pan et al., 2016). However, little is known about their osteogenic ability when they were employed as seed cells for bone defects repair. Therefore, it is highly desirable and a great challenge to investigate their osteogenic potential, enriching the stem cell-based therapeutic strategies for bone tissue regeneration.

To date, the compounds with capability to promote cell differentiation have been investigated in different bio-fields (Huang C et al., 2018; Pei et al., 2020; Wang et al., 2021). While, there are some application issues for the compounds in bio-fields, such as the tanglesome extraction process, instability of bio-activity, and bio-toxicity (Gu et al., 2021; Yang et al., 2021; Hu et al., 2022). Comparing to the compounds, nanomaterials have attracted considerable attention in the bio-field due to their distinct physicochemical properties, superior biocompatibility, and manipulation of stem cell fate (Zhang et al., 2021b; Huang et al., 2021). Recent studies suggested that magnetic iron oxide nanoparticles (IONPs) demonstrated great potential for versatile biomedical applications, especially stem cell therapy and bone tissue engineering (Hu et al., 2018; Soto et al., 2021). IONPs can facilitate osteogenic differentiation of stem cells *via* supporting transduction of dynamic mechanical stimulation, which is of great requirement for bone tissue regeneration (Henstock et al., 2014). Our previous study suggested that IONPs could promote osteogenic differentiation of human bone-derived mesenchymal stem cells (hBMSCs), and mechanism exploration *via* gene microarray assay and bioinformatics analysis exhibited that IONPs could activate the mitogen-activated protein kinase (MAPK) signal pathway (Wang et al., 2016). Incorporation of IONPs could also endow current bone repair scaffolds fabricated by electrostatic spinning and 3D-printing scaffolds with enhanced osteogenic performance both *in vitro* and *in vivo*. Hence, IONPs may endow PCSCs with great osteogenic bioactivity and subsequently used as seed cells for bone tissue regeneration.

To load the nanomaterials to achieve the excellent performance, the scaffolds and polymer matrices have been fabricated by various technologies (Huang et al., 2020; Liu et al., 2021; Xu et al., 2021). Scaffold materials have attracted considerable attention in stem cell-based therapeutic strategies for bone tissue regeneration due to their ability to hold stem cells for several cellular functions such as cell attachment, proliferation, and differentiation (Chen et al., 2019). There are numerous scaffolds, including hydrogels, acellular tissue matrix, and collagen, which have been used in bone tissue regeneration. Among these materials, hydrogels have been extensively utilized as bone repair scaffolds due to their good biocompatibility, favorable mechanical properties, suitable degradation rate, and superior biological activities (e.g., osteoconductivity and osteoinductivity) (Li et al., 2020). Hydrogels have the ability to hold and retain stem cells at the bone repair site, adaptively fill the lesion cavity, optimize the microenvironment, and mediate the directional growth of stem cells (Bai et al., 2018). Particularly, the physicochemical properties of hydrogels can be adjusted by varying the component and crosslinking methods. Recently, interpenetrating polymeric network (IPN) hydrogels with two or more crosslinked polymers are considered as a simple and easily feasible route to improve cell spreading and proliferation inside hydrogels (Geng et al., 2012). Therefore, IONPs-labeled PCSCs-incorporated IPN hydrogels could be used as promising repair scaffolds for bone tissue regeneration.

In the present study, PCSCs were successfully isolated and identified through immunohistochemically staining using anti-FGFR-3 antibodies. Subsequently, the effects of IONPs on biocompatibility and osteogenic differentiation of PCSCs were comprehensively investigated. Moreover, novel IPN hydrogel was employed as scaffold for holding IONPs-labeled PCSCs for *in vivo* bone regeneration assessment. Together with the fact that the IPN hydrogel was printable and could be used for 3D printing, we believed that IONPs-labeled PCSCs with enhanced osteogenic bioactivity could enrich the stem cell-based therapeutic strategies for bone tissue regeneration.

## 2 Materials and Methods

### 2.1 Materials and Reagents

Collagenase type I, sodium alginate, methacrylic anhydride, and 2-hydroxy4′-(2-hydroxyethoxy)-2-methylpropiophenone (Irgacure 2959, 98%) were obtained from Sigma-Aldrich Co. Ltd. (MO, United States). 4-arm poly (ethylene glycol) acrylate was provided by Sinopeg Biotech Co. Ltd. (China). Cell Counting Kit-8 was from Bimake (United States). Ultrapure water was achieved from a Millipore auto-pure system. All regents were used without further purification.

### 2.2 Culture and Identifying Precartilaginous Stem Cells

PCSCs were isolated individually from the ring of La-Croix extracted for polydactylism patient (age range 0.5–1 year) by collagenase type I (Zhang et al., 2020), which was approved by the ethics committee of Children’s Hospital affiliated to the Nanjing Medical University. Briefly, the ring of La-Croix, encompassing the lucent epiphyseal disk of embryo limbs, was precisely dissected under the operating microscope, followed by washing with phosphate-buffered saline (PBS) for three times. After that, the tissue was cut into fragments (1 mm×1 mm) digested with collagenase type I (1 mg/ml) for 12 h at 37°C in 5% CO_2_. After filtered by a 100-mesh aperture sieve, the obtained PCSCs were cultured in growth medium at 37°C in 5% CO_2_. The obtained PCSCs (passages 2 and 6) were used in subsequent experiments. For cells identification, the PCSCs were seeded into a 6-well plate containing poly-l-lysine-coated cover slips at a density of 5*10^4^ per well. After 3 days culture, the slips were immunohistochemically stained using anti-FGFR-3 antibodies [a specific marker for precartilaginous stem cells (Robinson et al., 1999)].

### 2.3 Iron Oxide Nanoparticles Synthesis and Characterization

IONPs were prepared according to a classic chemical co-precipitation method using polyglucose-sorbitol-carboxymethyl ether (PSC) as a stabilizer (Zheng L et al., 2021). Briefly, PSC (100 mg) was dissolved in 5 ml ultrapure water and the mixed solution was purified by argon for at least 5 min to remove the oxygen. After that, FeCl_3_ (30 mg) and FeCl_2_ (15 mg) were dissolved in 10 ml ultrapure water and the mixed solution was added to the reaction system, followed by the addition of ammonium hydroxide (500 mg, 28% w/v) under vigorous stirring at 80°C for 30 min. The obtained IONPs were collected and dialyzed using membrane tubing (MWCO = 3000) to remove the free PSC. As for characterization, the morphology of IONPs was characterized by transmission electron microscopy (TEM, JEOL 1200EX). The hydrodynamic size of IONPs was measured by dynamic light scattering (DLS) (Malvern Zetasizer Nano ZS90, United Kingdom).

### 2.4 Cell Experiment

#### 2.4.1 Cell Culture

The PCSCs were cultured at 37°C with 5% CO_2_ in growth medium (DMEM medium containing 10% fetal bovine serum and 1% penicillin/streptomycin). For osteogenic induction, the culture medium was replaced by osteogenic medium (growth medium supplemented with 0.1 μM dexamethasone, 50 μg/ml ascorbic acid, and 10 mM β-glycerophosphate). The culture medium was replaced every 3 days.

#### 2.4.2 Cellular Uptake Observation of Iron Oxide Nanoparticles by Prussian Blue Staining

After seeded in 24-well plates (1*10^5^ cells per well) and cultured for 24 h, the PCSCs were incubated with various amount of IONPs (Fe concentration: 50, 100, and 200 mg/ml) for 24 h, collected, and fixed with 4% (v/v) formaldehyde (PFA) and a Perl’s blue staining assay was performed to determine the internalization of IONPs. After that, the cells were observed by an inverted optical microscope (Olympus IMT-2, Tokyo, Japan).

#### 2.4.3 Cell Viability

The toxicity of IONPs toward PCSCs was assessed using a standard Cell Counting Kit-8 (CCK-8) assay (Zhang et al., 2021a). In brief, PCSCs were seeded in 96-well plates (1*10^4^ cells per well) before various amount of IONPs (Fe concentration: 50, 100, and 200 mg/ml) were added. To test the cytotoxicity, 10 μl CCK-8 was added and cultured for another 1 h. Cell viability was detected according to the OD value observed by a microplate reader (Multiskan GO, Thermo Fisher Scientific, United Ststes).

#### 2.4.4 Live/Dead Staining

After treated with IONPs (100 mg/ml) for 3 days, PCSCs were harvested, washed with PBS for three times, and stained with calcein-AM/PI (Solarbio, China). Fluorescence images were observed using an inverted optical microscope (Olympus IMT-2, Tokyo, Japan).

#### 2.4.5 Alkaline Phosphatase and Alizarin Red S Staining

PCSCs treated with IONPs (100 mg/ml) were harvested after osteogenic induction for 14 days (ALP staining) and 21 days (ARS staining). The cells were stained using the BCIP/NBT alkaline phosphatase color development kit (Beyotime, China) and 5% ARS staining solution (Sigma, United Ststes) according to the manufacturer’s instructions, respectively, followed by the observation using an inverted optical microscope (Olympus IMT-2, Tokyo, Japan).

#### 2.4.6 Real-Time Quantitative PCR

PCSCs were cultured in a 6-well plate (3*10^5^ cells per well) for 24 h, followed by the treatment of IONPs (100 mg/ml) for 3 days. After that, total RNA was isolated using an RNA-Quick Purification Kit (Yishan Biotech, Shanghai, China) and cDNA was generated using a HiScript II Q RT SuperMix according to the manufacturer’s instructions. Finally, the quantitative PCR was detected using a ChamQTM SYBR Color qPCR Master Mix (Vazyme Biotech). Supplementary Table S1 listed the forward and reverse primers of the investigated osteogenic-related genes.

### 2.5 Preparation of Interpenetrating Polymeric Network Hydrogel for Holding Precartilaginous Stem Cells

An interpenetrating polymeric network based on photo-crosslinking of methacrylated alginate and 4-arm poly (ethylene glycol)-acrylate (4A-PEGAcr) was constructed for holding PCSCs, where methacrylated alginate was synthesized according to a reported study (Araiza-Verduzco et al., 2020). In brief, 100 mg methacrylated alginate and 50 mg 4A-PEGAcr were dissolved in 5 ml PBS at 37°C for 1 h, followed by the addition of I2959 (1% (w/v)). After that, PCSCs were already incorporated with IONPs *in vitro* to acquire great osteogenic activity, followed by the transfer 500 μl of the mixed solution containing 1*10^6^ IONPs-incorporated PCSCs to a flat-bottomed 1-ml tube and subjected UV-light irradiation (365 nm) for 90 s. The as-prepared PCSCs-incorporated hydrogel was submerged in PBS at 37°C for 1 h and implanted to femur condyle defect of rabbit for *in vivo* bone regeneration assessment.

### 2.6 Animal Experiment

All experimental protocols were approved by the ethics committee of Drum Tower Hospital affiliated to the Medical School of Nanjing University, and performed according to the Institutional Animal Care and Use Committee (IACUC) guidelines.

#### 2.6 1 The Femur Condyle Defect Model

A total of 12 male Newland rabbits (3.5 kg) were enrolled in the study and randomly divided into three groups (control group, neat hydrogel group, and PCSCs-hydrogels group). Bilateral femur condyle defect (high: 3 mm, diameter: 5 mm) was made, followed by the implantation of hydrogels and the defects in the control group remained blank. All rabbits were sacrificed at week 12 post-operation for *in vivo* bone regeneration assessment.

#### 2.6.2 Micro-CT Analysis

The high-resolution micro-CT scanner (Scanco Medical, Switzerland) was used to evaluate the *in vivo* bone regeneration, where bone mineral density (BMD), bone volume/total volume (BV/TV), trabecular number (TB.N), trabecular separation (TB.Sp), and trabecular thickness (TB.Th) were quantified. A commercial software MIMICS19.0 (Materialise, Leuven, Belgium) was used to generate 3D models of the harvested femurs.

#### 2.6.3 Histological Analysis

After fixed with formalin at 4°C for 24 h, the harvested femur condyles were decalcified using 15% ethylene diamine tetra-acetic acid (EDTA) for 28 days. After that, the decalcified femur condyles were embedded in paraffin and sectioned at 5 μm of hematoxylin and eosin (H&E) and Masson’s trichrome staining. The histological analysis of major organs (heart, liver, spleen, lung, and kidney) followed the same process except for the decalcification process.

### 2.7 Statistical Analysis

All experiments were performed with three replicates unless otherwise stated. Data are mean ± standard deviation. Statistical analysis was performed with Origin software (8.5 version). Asterisks in statistical analysis indicate statistically significant differences between the control and experimental groups (∗*p* < 0.05; ∗∗*p* < 0.01; and ∗∗∗*p* < 0.005).

## 3 Results

### 3.1 Isolation and Identification of Precartilaginous Stem Cells

**FIGURE 1:**
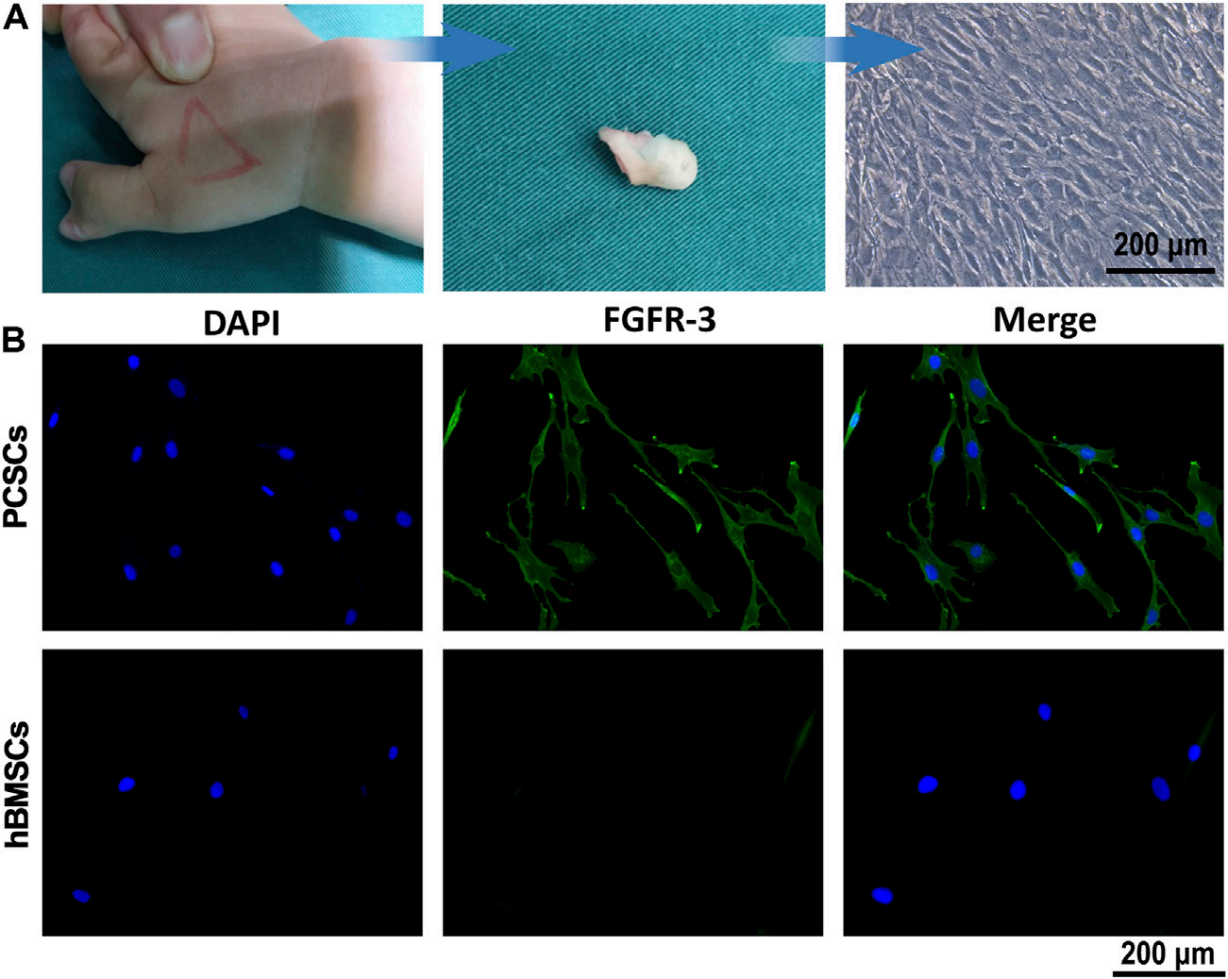
Isolation and identification of PCSCs. **(A)** Process of isolation of PCSCs from the ring of La-Croix extracted from polydactylism patient. **(B)** Representative images of immunostaining for DAPI (blue) and FGFR-3 (green), and hBMSCs were used as control.

PCSCs were isolated individually from the ring of La-Croix extracted for polydactylism patient (age range 0.5–3 years) by collagenase type I, the process of which was shown in Figure 1A. It could be seen that the PCSCs grow well under a light microscope. In addition, fibroblast growth factor receptor 3 (FGFR-3), a specific marker for precartilaginous stem cells was used to identify the PCSCs (Robinson et al., 1999), where human bone marrow-derived mesenchymal stem cells (hBMSCs) were used as control. As shown in Figure 1B, the immunofluorescence staining results for PCSCs demonstrated the positive expression of FGFR-3. In contrast, almost no FGFR-3-positive expression was found in hBMSCs (Figure 1C). These results unarguably confirmed that the isolated cells were PCSCs, which could be used for further *in vitro* osteogenic differentiation and *in vivo* bone regeneration assessment.

### 3.2 Preparation and Characterization of Iron Oxide Nanoparticles

**FIGURE 2:**
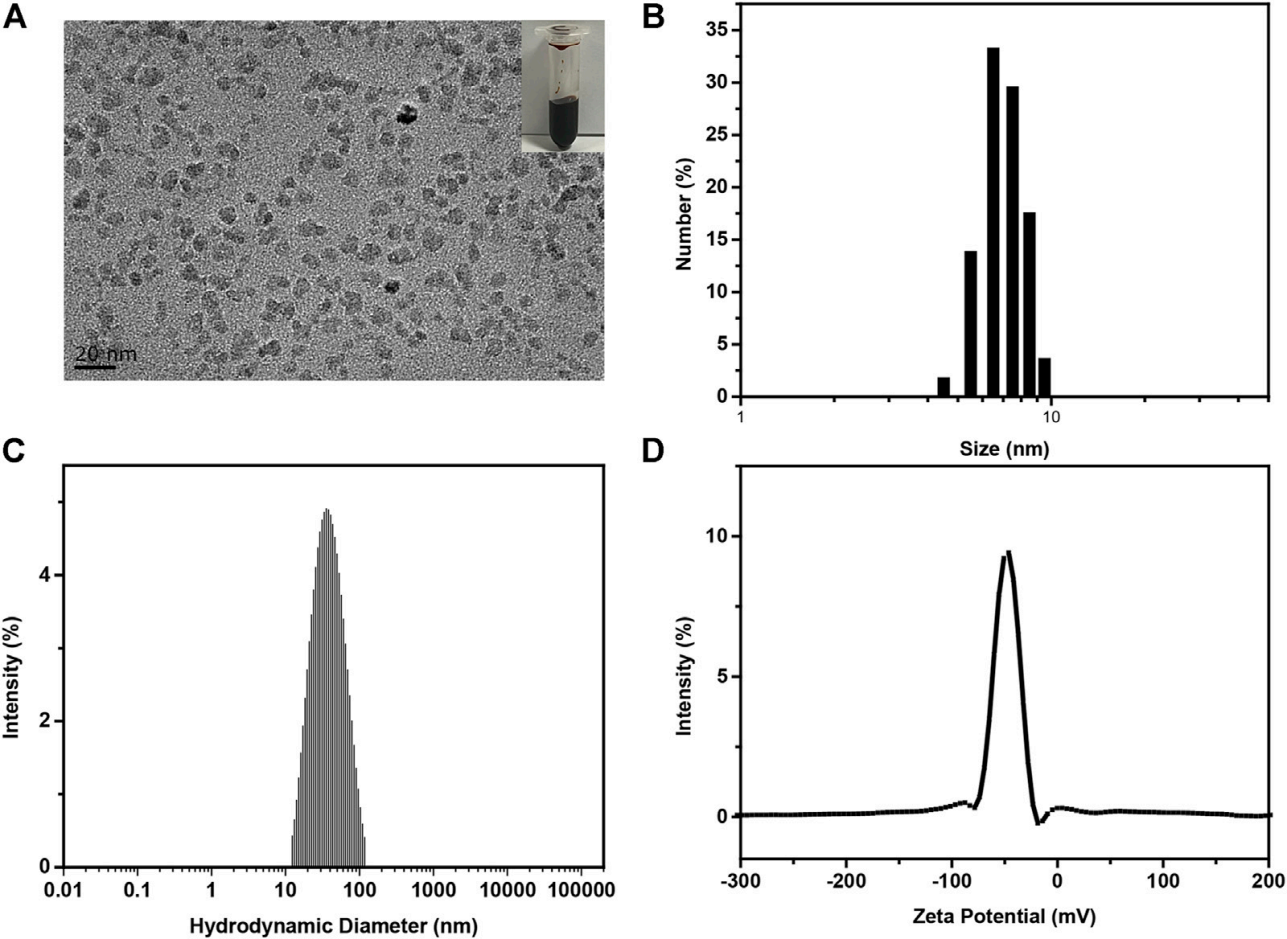
Preparation and characterization of IONPs. **(A)** Typical TEM image, **(B)** statistical size distribution according to **(A**–**C)** hydrodynamic diameter, **(D)** zeta potential of as-prepared PSC-coated IONPs.

IONPs were synthesized by a classic chemical co-precipitation method (Chen et al., 2018). The as-prepared IONPs solution displayed dark brown color (Figure 2A, inset). Characterizations of IONPs were performed using TEM and DLS. TEM images (Figure 2A) demonstrated that the obtained IONPs exhibited dimensional homogeneity and excellent dispersity. The statistical average size (Figure 2B) of the iron oxide cores were 7.14 ± 0.68 nm. In addition, the hydrodynamic size (Figure 2C) of IONPs was 34.9 nm, the value of which was larger than their physical size due to the hydrated PSC shell. The polydispersity index (P.I.) was 0.229, indicating the excellent monodispersity. It was also demonstrated that the zeta potential (Figure 2D) of IONPs was −41.69 mV, and the negatively charged value was attributed to the numerous carboxyl of PSC (Yu et al., 2020). It should be mentioned that the synthetic route of PSC-coated IONPs exactly followed the technology of ferumoxytol, which is the only inorganic nano-drug approved by the Food and Drug Administration (FDA) for clinical applications. Hence, IONPs we used in our study were of great biosecurity, potentially translatable, and could be used to investigate their biological effects on PCSCs.

### 3.3 *In Vitro* Biocompatibility and Cellular Uptake of Iron Oxide Nanoparticles on Precartilaginous Stem Cells

**FIGURE 3:**
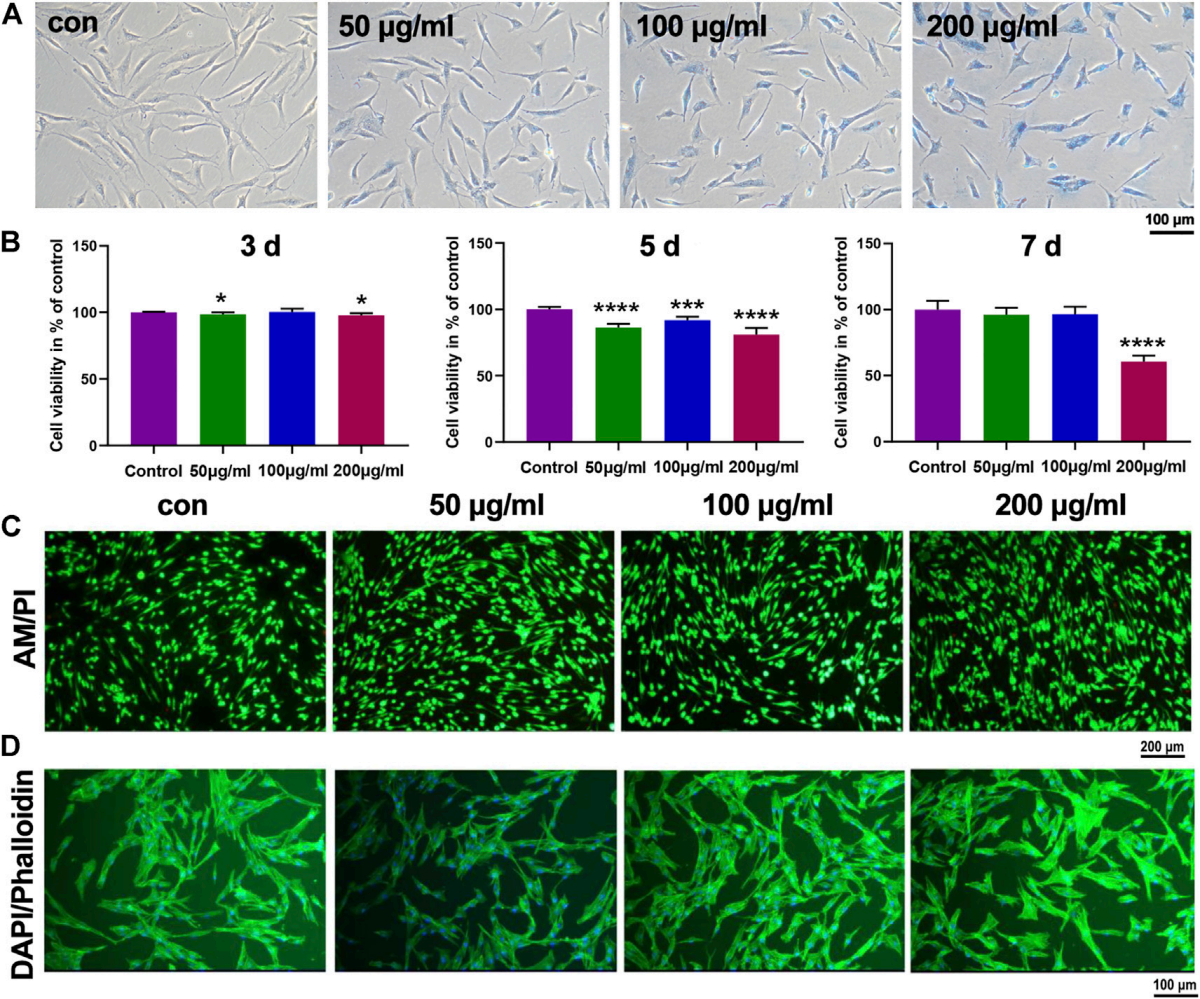
*In vitro* biocompatibility and cellular uptake of IONPs on PCSCs. **(A)** Prussian blue staining of PCSCs treated with IONPs with various concentrations (50, 100, and 200 μg/ml). **(B)** Cell viability of PCSCs after incubated with IONPs (concentration: 50, 100, and 200 μg/ml) for 3, 5, and 7 days. Representative scan of **(C)** live/dead staining (green- and red-labeled cells denote living and dead cells, respectively) and **(D)** cytoskeleton staining of PCSCs after treated with IONPs with various concentration (50, 100, and 200 μg/ml), respectively. Asterisk indicates statistically significant differences between the control and experience groups (∗*p* < 0.05; ∗∗∗*p* < 0.005; and ∗∗∗∗*p* < 0.001).

It has been widely reported that the cytomembrane of biological cells is negatively charged due to the hydrophilic phosphonyl group of phospholipid bilayer (Huang B et al., 2018). Hence, the negatively charged PSC-coated IONPs might hardly be taken by cells, limiting their interactions with biological cells. Hence, we employed positively charged poly-L-lysine to modify IONPs using a reported method (Sun et al., 2021). Effects of IONPs on cell viability and cellular uptake of PCSCs were first studied. As shown in Figure 3A, the Prussian blue staining results demonstrated that the cellular uptake of IONPs by PCSCs was obviously enhanced with the concentration of IONPs increased from 50 to 200 μg/ml. The biocompatibilities of IONPs with various concentrations toward PCSCs were further investigated using Cell Counting Kit-8 (CCK8) at 3, 5, and 7 days (Figure 3B). IONPs of all three concentrations demonstrated no obvious cytotoxicity toward PCSCs after 3 and 5 days’ treatment with the viability exceeding 80%. However, after treated with IONPs (200 μg/ml) for 7 days, obvious decrease of cell viability could be observed, resulting in the apparent toxicity. Hence, 100 μg/ml could be considered as a proper concentration for further osteogenic assessment. To further verify their potential toxicity to PCSCs, live/dead staining was employed, and the results were exhibited in Figure 3C. Nearly all the PCSCs were alive (green fluorescence) after treated with IONPs at all investigated concentrations. The effect of IONPs on attachment of PCSCs was assessed using phalloidine/DAPI staining (Figure 3D), where it could be seen that PCSCs spread out with their filopodia extended. These results indicated that IONPs were biocompatible toward PCSCs and could be used to further assess their biological activity.

### 3.4 Effects of Iron Oxide Nanoparticles on Osteogenic Differentiation of Precartilaginous Stem Cells

**FIGURE 4:**
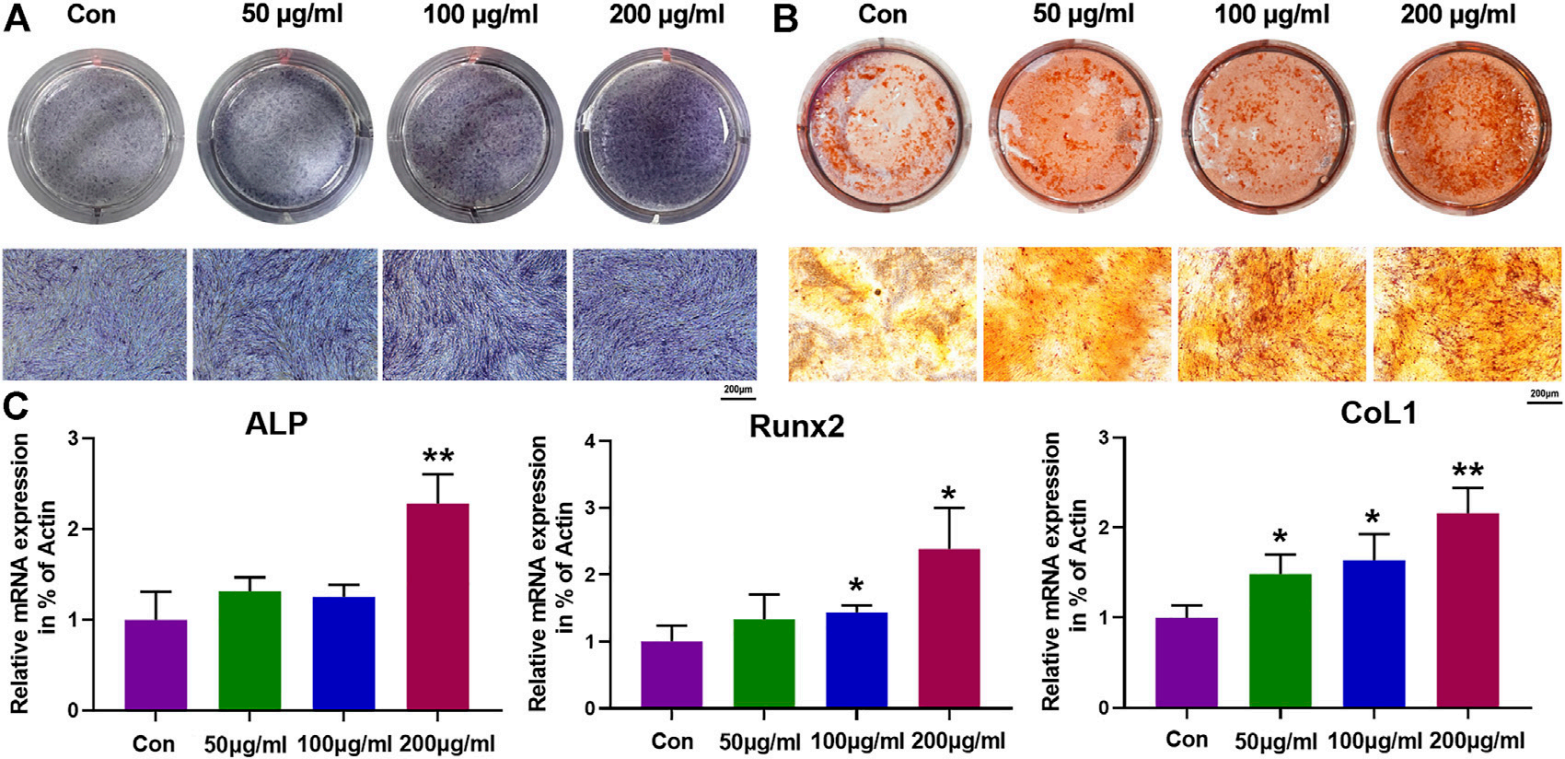
Effects of IONPs on osteogenic differentiation of PCSCs. **(A)** ALP staining of PCSCs after treated with various concentrations of IONPs (50, 100, and, 200 μg/ml) for 14 days. **(B)** ARS staining of PCSCs after treated with various concentrations of IONPs (50, 100, and 200 μg/ml) for 28 days. **(C)** Real-time PCR analysis of ALP, Runx-2, and COL1 expression of PCSCs after treated with various concentrations of IONPs (50, 100, and 200 μg/ml) for 3 days. Asterisk indicates statistically significant differences between the control and experience groups (∗*p* < 0.05 and ∗∗*p* < 0.01).

Osteogenic differentiation is a crucial process for biological cells when it was used as a osteogenic bioactive agent for bone tissue regeneration, where alkaline phosphatase (ALP) activity and formation of mineralized nodule are two significant signals for the early and final stages process of osteogenic differentiation, respectively (Zhang et al., 2017). Hence, the effects of IONPs on osteogenic differentiation of PCSCs were further assessed using ALP and alizarin red S (ARS) staining. As shown in Figure 4A,B, IONPs demonstrated a dose-dependent effect in increasing ALP activity and mineralized nodule formation. In addition, the expression of osteogenic-related genes, including ALP, runt-related transcription factor 2 (Runx2), and collagen type 1 (COL1) were determined using real-time PCR in PCSCs treated with IONPs (100 μg/ml) for 3 days. As illuminated in Figure 4C, the expression of these osteogenic-related genes were significantly upregulated after treated with IONPs, where high concentration IONPs (200 μg/ml) exhibited the strongest effect on accelerating the osteogenic-related genes expression of PCSCs. Taken together, these results confirmed that IONPs showed the positive effect on facilitating osteogenic differentiation of PCSCs.

### 3.5 Preparation and Characterization of Precartilaginous Stem Cells-Hydrogels

**FIGURE 5:**
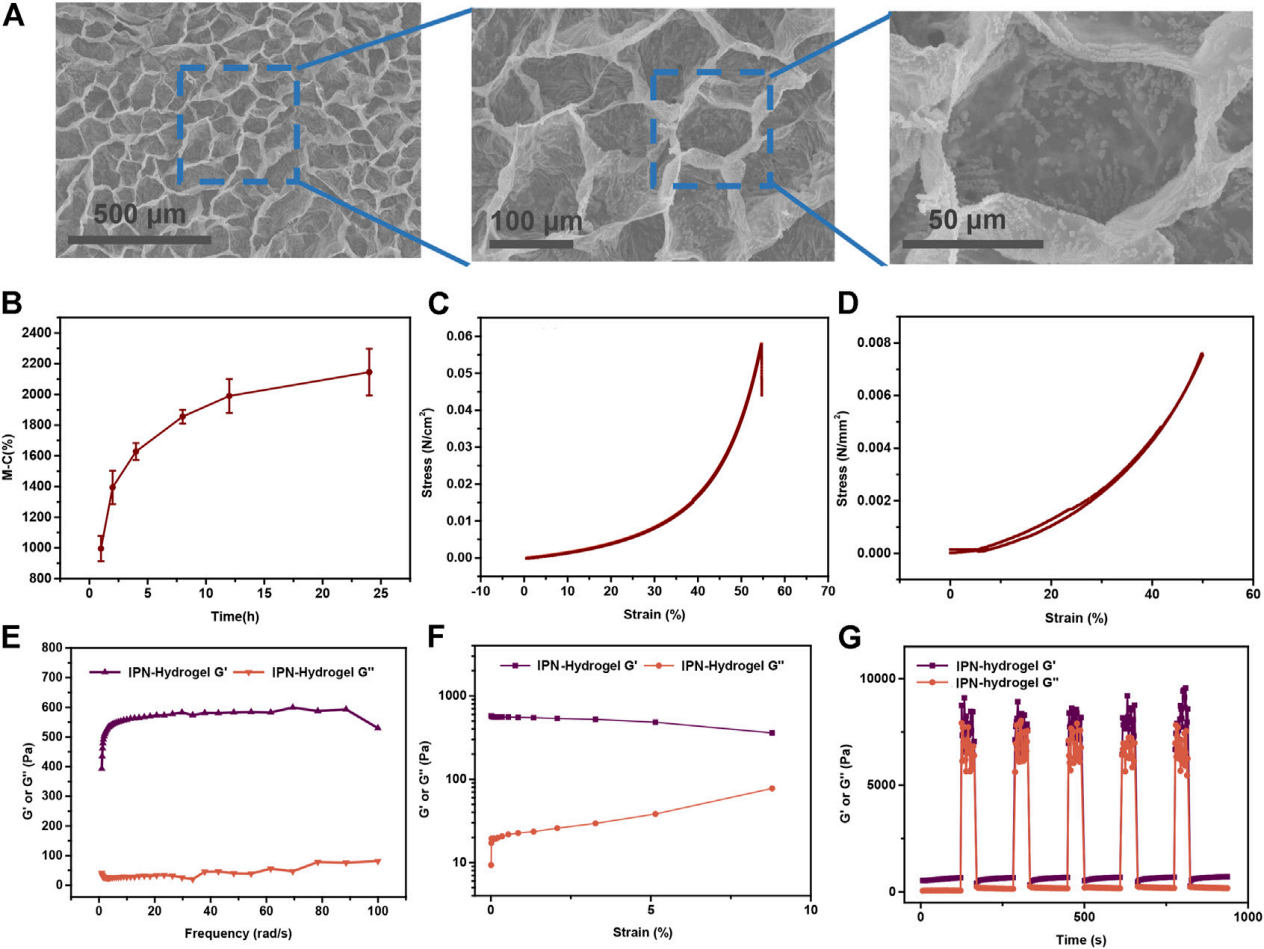
Preparation and characterization of PCSCs-hydrogels. **(A)** Surface topography and their local magnification of lyophilized PCSCs-hydrogels observed by SEM. **(B)** Swelling rate at different time from 1 to 24 h. **(C)** Uniaxial stress–strain curves under compression until cracking. **(D)** Uniaxial compression–relaxation curves. **(E)** G′ and G″ of hydrogels measured in a strain sweep experiment (from 0.1 to 10% strain, 6.28 rad s-1) at room temperature. **(F)** G′ and G″ of the hydrogels measured in a frequency sweep experiment (from 0.1 to 60 rad s-1, 1% strain) at room temperature. **(G)** Hydrogels in a destroy–recovery experiment at room temperature. The frequency and strain were set to the frequency of 100 Hz and amplitude of 300% to destroy the coordination interactions and switched back to a frequency of 6.28 rad s-1 and amplitude of 0.1% to monitor recovery of the mechanical properties.

To further investigate the *in vivo* bone regeneration ability of IONPs-labeled PCSCs when used as a osteogenic bioactive agent, smart scaffolds with several advantages, including excellent biocompatibility, porous microstructures, and appropriate mechanical properties should be used for holding PCSCs (Zheng J et al., 2021). Among these scaffolds, hydrogels with three-dimensional culture matrices have been widely used as space filling agents (flexibility in fitting in any application site) and delivery vehicles for bioactive molecules (controllability pore size in the polymer network) (Ma et al., 2021). In our study, novel biomimetic interpenetrating polymeric network (IPN) hydrogel constructed by methacrylated alginate (MLA) and 4-arm poly (ethylene glycol)-acrylate (4A-PEGAcr) through photo-crosslinking upon exposure to long-wave UV light was used as scaffold to hold PCSCs for bone regeneration assessment. The morphology of lyophilized IPN hydrogel was characterized by SEM and the results are shown in Figure 5A. It could be seen that our prepared IPN hydrogel demonstrated a porous microstructure with a pore size of around 30–50 nm, facilitating several cellular functions such as cell attachment, proliferation, and differentiation (Ajdary et al., 2021). Moreover, physicochemical properties of IPN hydrogel, including swelling rate, mechanical, and rheological properties were further measured. As shown in Figure 5B, the IPN hydrogel swelled to its maximum swelling ratio of about 2100% within 24 h. The excellent swelling property might make our hydrogel suitable for *in vivo* applications. The compressive mechanical properties of our as-prepared hydrogel were detected using standard mechanical tests. According to the results of compression-crack test (Figure 5C) and compression-relaxation cycles test (Figure 5D), the compressive modulus of IPN hydrogel was 210 Pa and the gels can compress a strain of more than 55%, indicating excellent load-bearing ability. In addition, only slight energy dissipation could be observed, suggesting that the as-prepared exhibited reliable mechanical properties. In addition, rheological properties were further measured and the results of frequency-sweep test, strain-sweep test, and step-strain test are demonstrated in Figures 5E–G, respectively. It could be seen that as-prepared IPN hydrogel demonstrated a solid-like behavior [storage modulus (G′) was larger than the loss modulus (G″)] and dominant elastic property (partly destroyed and subsequently completely recovered). All these features suggested that our IPN hydrogel with excellent physicochemical properties could be employed as scaffold for holding IONPs-labeled PCSCs for bone tissue regeneration.

### 3.6 *In Vivo* Bone Regeneration Assessment of Iron Oxide Nanoparticles-Labeled Precartilaginous Stem Cells-Hydrogels

**FIGURE 6:**
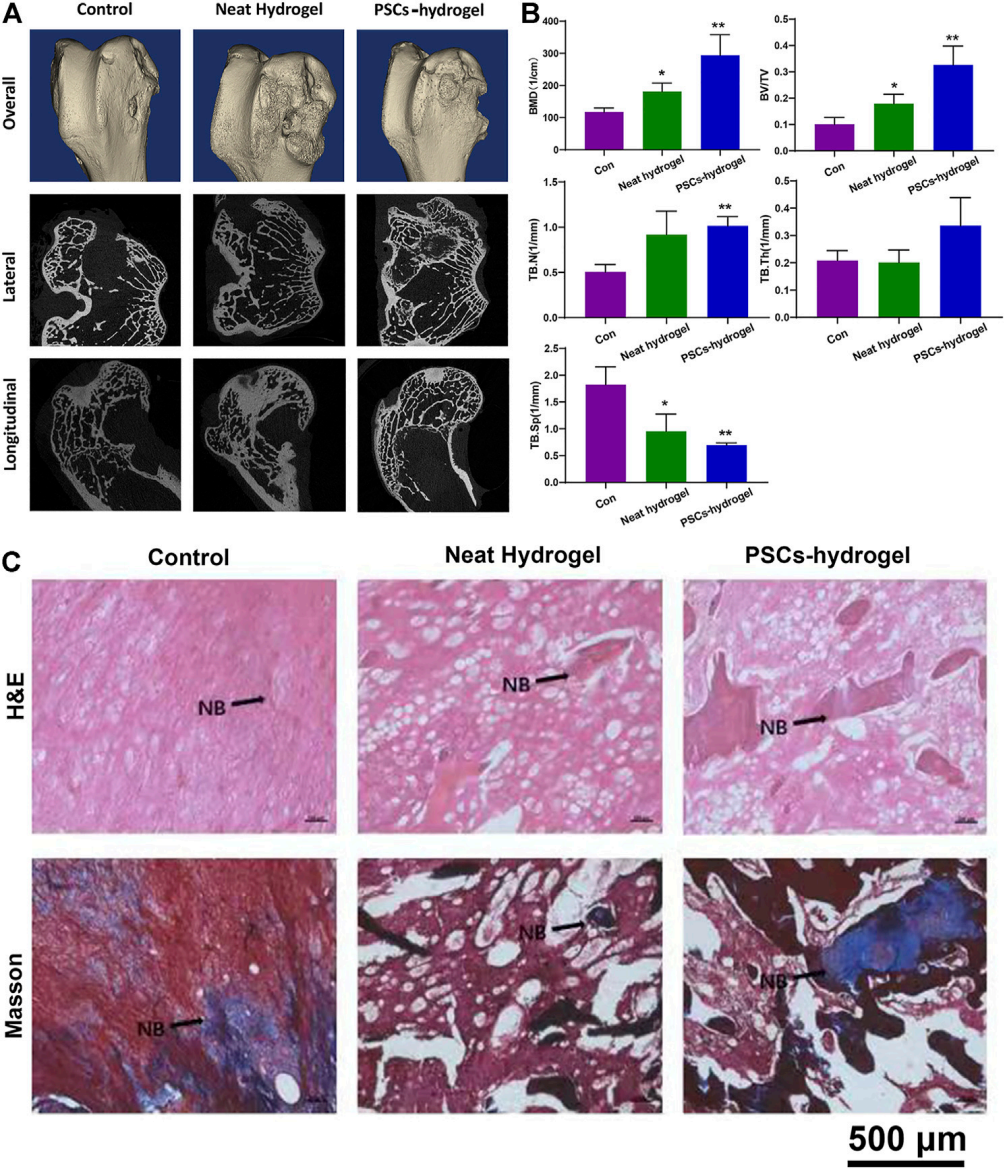
*In vivo* bone regeneration assessment of PCSCs-hydrogels. **(A)** Reconstructed 3D micro-CT and sectional images of femoral condyle after treated with PCSCs-hydrogels for 12 weeks. **(B)** Quantitative evaluation of the regenerated area by analyzing the parameter of micro-CT: BMD, BV/TV, TBN, TB.Sp, and TB.Th. **(C)** H&E and Masson staining images of bone defect implanted with neat hydrogel and PCSCs-hydrogel. Abbreviation: NB, newly formed bone. Asterisk indicates statistically significant differences between the control and experience groups (∗*p* < 0.05 and ∗∗*p* < 0.01).

*In vivo* bone regeneration performance of IONPs-labeled PCSCs were finally assessed using a rabbit model of 5 mm femoral defect, where IPN hydrogel was used as scaffold for holding PCSCs. IONPs-labeled PCSCs-hydrogels (height: 5 mm and diameter: 5 mm) were directly implanted into the femur defects of rabbits and neat IPN hydrogels were used as control. After 3 months of implantation, femurs of rabbits were harvested and comprehensively characterized by micro-CT and histological analysis. Micro-CT data (Figure 6A), including the reconstructed three-dimensional (3D) model, lateral and longitudinal sectional images exhibited that more newly formed bone could be observed around the bone defect area in the PCSCs-hydrogels group compared with that in the neat hydrogel and control groups. Furthermore, quantitative bone density analysis of newly formed bone, including bone mineral density (BMD), bone volume per total volume (BV/TV), and the trabecular parameters of the cancellous bone such as trabecular thickness (TB.Th), trabecular number (TB.N), and trabecular spacing (TB.Sp) were performed according to the micro-CT data. As demonstrated in Figure 6B, the values of BMD and BV/TV were significantly increased in the PCSCs-hydrogels group compared with a neat hydrogel group, indicating that PCSCs played an important role in accelerating bone formation. Further trabecular results exhibited the increased TB.Th and TB.N values and the decreased TB.Sp after treated with IONPs-labeled PCSCs-hydrogels by following the similar trend with the BMD. Furthermore, histological analysis (H&E and Masson staining) was also used to assess the bone regeneration performance. As shown in Figure 6C, more newly formed bone and trabecula could be observed in the PCSCs-hydrogel group compared with that in the neat hydrogel and control groups with only fibrotic connective tissues. Finally, the *in vivo* biosafety of our IONPs-labeled PCSCs-hydrogels was detected using histopathological analysis. Major organs, including heart, liver, spleen, lung, and kidney were collected from the rabbits that were implanted IONPs-labeled PCSCs-hydrogels and neat hydrogel for 3 months and histopathologically examined through hematoxylin and eosin (H&E) and Masson staining. As shown in Supplementary Figure S1, no obvious damage or inflammation of these tissue slices could be observed in all the investigated groups, indicating that both IPN hydrogels and IONPs-labeled PCSCs were of great biocompatibility.

## 4 Discussion

Stem cell-based therapeutic strategies are considered to be very promising platforms for bone tissue regeneration, among which PCSCs has been the focus of significant interest due to their potential regenerative capacity to transform themselves into a crowd of different cells (Luo et al., 2012). Although, PCSCs showed excellent chondrogenic ability but little is known about their osteogenic ability when they were employed as seed cells for bone defects repair. Recent investigations demonstrated that nanomaterials, especially IONPs could modulate the fate of stem cells and interactions between nanomaterials and stem cells have taken into serious consideration in facilitating stem cell-based therapeutic strategies for bone tissue regeneration (Wang et al., 2020). In this study, we have isolated PCSCs from the ring of La-Croix extracted for polydactylism patient and observed that IONPs could promote osteogenic differentiation of PCSCs. Subsequently, IONPs-labeled PCSCs-incorporated IPN hydrogel was extensively utilized as repair scaffolds for bone tissue regeneration and the results indicated that IONPs-labeled PCSCs could accelerate the bone repair process rapidly and efficiently.

There are two common methods to obtain somatic stem cells 1) directional differentiation of embryonic stem cells (ESC) (Wichterle et al., 2002) and 2) *in vitro* isolation and cultivation of the desired stem cells in specific tissue (Eberle et al., 2013). The method 1) has some limitations in terms of complex condition for directional differentiation of ESC. In our study, PCSCs were isolated individually and identified using FGFR-3. The positive expression of FGFR-3 unarguably confirmed that the isolated cells were PCSCs. Subsequently, IONPs were prepared by a classic chemical co-precipitation method and the obtained IONPs were of low polydispersion, which guaranteed the reproducibility of their biological effect.

To assess the bio-toxicity of IONPs, the viability and adhesion of PCSCs cultured with IONPs (50, 100, and 200 μg/ml) were detected. The results demonstrated that IONPs with the concentration lower than 100 μg/ml were non-toxic toward PCSCs, in agreement with previous reports (Wang et al., 2017). Based on the biocompatibility results, the effects of IONPs on osteogenic differentiation of PCSCs at concentrations of 50, 100, and 200 μg/ml. The ALP activity level (an important indicator for osteoblast differentiation) and formation of mineralized nodule were considered as two specific stages of osteogenic differentiation (Mohamed-Ahmed et al., 2018). In our study, after treated with IONPs (especially at a concentration of 200 μg/ml), the ALP activity level and mineralization were significantly enhanced, indicating the high osteogenic potential. The osteogenic-related genes, including ALP, Runx2, and COL1 were further detected to verify their osteogenic ability, where Runx2 was considered as an early master regulator for the initiation of osteogenesis that regulate the osteogenic-related genes such as COL1 and ALP (Komori, 2018). The results showed that all these genes were upregulated after treated with IONPs, which matched well with the aforementioned ALP activity level and mineralization results. Hence, IONPs could be used to induce osteogenic differentiation of PCSCs and IONPs-labeled PCSCs with enhanced osteogenic activity could be further used for *in vivo* bone tissue regeneration.

Smart biomaterials such as hydrogels, nanofibrils, and biological ceramics have proven incredibly beneficial as scaffold for bone tissue regeneration due to their adjustable nanostructure, excellent biocompatibility, biodegradability, proper mechanical properties, enhanced osseointeration capability and superior biological activities (Yue et al., 2015; Huang et al., 2020; De France et al., 2021). Among these biomaterials, hydrogels with additional advantages such as printability have attracted considerable attention. Numerous of materials have been used to construct bioactive hydrogels for bone tissue regeneration. Alginate, a naturally occurring biopolymer, has been widely used design materials for bone tissue regeneration due to their attractive properties such as biocompatibility, biodegradability, antibacterial activity, hydrophilicity, and nontoxicity (Hernández-González et al., 2020). Previous study demonstrated that alginate could be methacrylated and the obtained ionic and photo-crosslinked alginate-methacrylate hydrogels showed modulable mechanical properties (Araiza-Verduzco et al., 2020). PEG-based hydrogels with great printability have been the focus of significant interest in designing multifunctional scaffolds for bone tissue regeneration, where PGE was the common components for photo-crosslinked three-dimensional (3D) printing. In our study, MLA/4A-PEGAcr IPN hydrogel was prepared through photo-crosslinking and ionic-crosslinking. The as-prepared IPN hydrogel possessed porous nanostructure and proper mechanical properties and could be used to incorporate IONPs-labeled PCSCs for bone tissue regeneration assessment. As for the *in vitro* results, both micro-CT and histological analysis indicated that the implantation of IONPs-labeled PCSCs significantly accelerated the process of bone formation, indicating that IONPs-labeled PCSCs could endow current bone repair scaffolds with excellent osteogenic activity.

## 5 Summary

This study is the first report of osteogenesis of PCSCs induced by IONPs, which is evidenced by an enhanced ALP activity level, mineralized matrix nodules, and osteogenic-related gene expressions. Further *in vivo* therapeutic performance of bone defect repair could be obtained by incorporation of IPN hydrogel as scaffold. Together with the fact that the IONPs-labeled PCSCs-incorporated IPN hydrogel was of great printability, biosafety, and improvement of cell spreading and proliferation, we believed that this PCSCs-based scaffolds could enrich the stem cell-based therapeutic strategies for bone tissue regeneration.

## Data Availability

The original contributions presented in the study are included in the article/Supplementary Material, further inquiries can be directed to the corresponding authors.
