# Supplementary material for: Osteogenesis of Iron Oxide Nanoparticles-Labeled Human Precartilaginous Stem Cells in Interpenetrating Network Printable Hydrogel
Source: Front Bioeng Biotechnol. 2022 Apr 29;10:872149. doi: 10.3389/fbioe.2022.872149 (PMC9099245; doi:10.3389/fbioe.2022.872149)
Supplement: Supplementary file 1 [file Table1.DOCX]

Supplementary Material

**
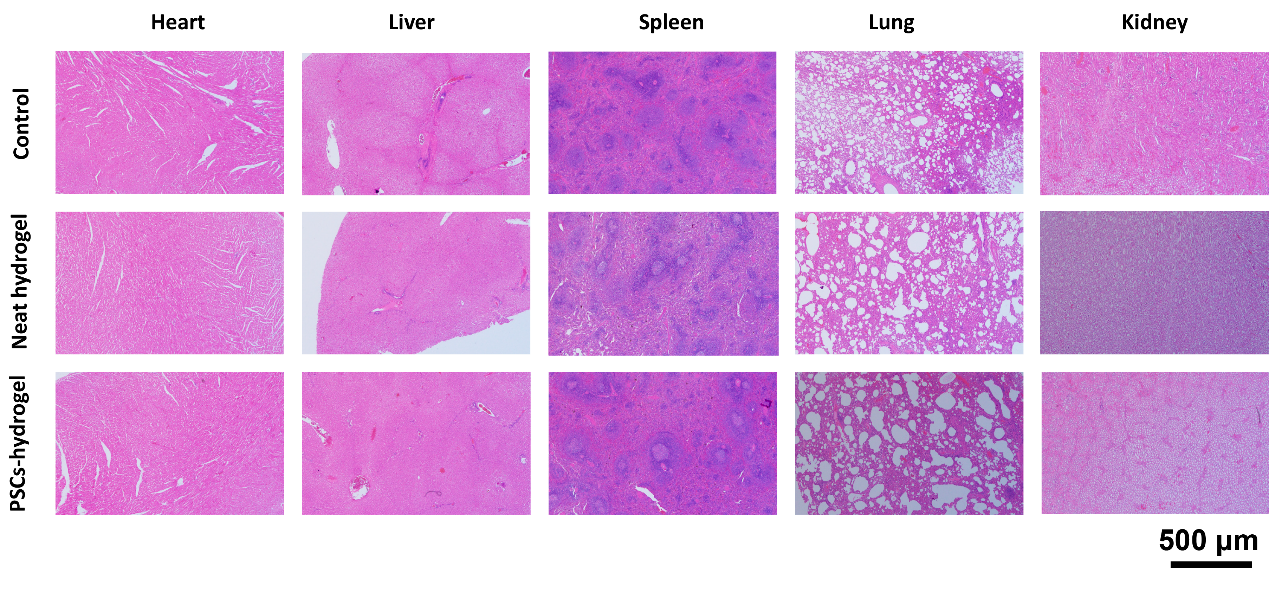
**

**Figure S1.** Histological examination of major organs collected from neat hydrogel and PCSCs-hydrogels administrated rabbits.

**Table S1**. Primer sequence used for quantitive PCR

| Primer | Forward | Reverse |
| --- | --- | --- |
| GAPDH | AGGTCGGTGTGAACGGATTTG | GGGGTCGTTGATGGCAACA |
| Runx2 | GACTGTGGTTACCGTCATGGC | ACTTGGTTTTTCATAACAGCGGA |
| ALP | CCAACTCTTTTGTGCCAGAGA | GGCTACATTGGTGTTGAGCTTTT |
| COL-I | TCGTGCCTAGCAACATGCC | TTTGTCAGAATACTGAGCAGCAA |
